# Carbon Nanotube-Modified Nickel Hydroxide as Cathode Materials for High-Performance Li-S Batteries

**DOI:** 10.3390/nano12050886

**Published:** 2022-03-07

**Authors:** Qianwen Jin, Yajing Yan, Chenchen Hu, Yongguang Zhang, Xi Wang, Chunyong Liang

**Affiliations:** 1 State Key Laboratory of Reliability and Intelligence of Electrical Equipment, School of Materials Science and Engineering, Hebei University of Technology, Tianjin 300130, China; jinqianwen111@163.com (Q.J.); yajingy@126.com (Y.Y.); h15364966537@163.com (C.H.); 2 China Center for Information Industry Development, Beijing 100048, China

**Keywords:** Li-S battery, carbon nanotube, nickel hydroxide, cathode

## Abstract

The advantages of high energy density and low cost make lithium–sulfur batteries one of the most promising candidates for next-generation energy storage systems. However, the electrical insulativity of sulfur and the serious shuttle effect of lithium polysulfides (LiPSs) still impedes its further development. In this regard, a uniform hollow mesoporous Ni(OH)_2_@CNT microsphere was developed to address these issues. The SEM images show the Ni(OH)_2_ delivers an average size of about 5 μm, which is composed of nanosheets. The designed Ni(OH)_2_@CNT contains transition metal cations and interlayer anions, featuring the unique 3D spheroidal flower structure, decent porosity, and large surface area, which is highly conducive to conversion systems and electrochemical energy storage. As a result, the as-fabricated Li-S battery delivers the reversible capacity of 652 mAh g^−1^ after 400 cycles, demonstrating excellent capacity retention with a low average capacity loss of only 0.081% per cycle at 1 C. This work has shown that the Ni(OH)_2_@CNT sulfur host prepared by hydrothermal embraces delivers strong physical absorption as well as chemical affinity.

## 1. Introduction

Lithium–ion batteries (LIBs), commercialized since the 1990s, have been leading the secondary battery market [1,2]. However, the development of LIBs is constrained by their limited theoretical energy density [3,4,5]. The booming Li-S battery has emerged as the most prospective battery due to its salient theoretical specific capacities of lithium and sulfur [6,7]. Elemental sulfur as a low-cost and non-toxic material, theoretically offers a high capacity of 1675 mAh g^−1^ [8,9]. Despite the numerous advantages, its commercialization path for lithium-sulfur batteries is still hindered by certain intrinsic factors [10,11,12]. First, sulfur and sulfides have poor electronic conductivity, resulting in low active materials utilization and specific capacity [13]. Second, a great structure and volume change (~80%) in the process of cycling causes hidden dangers for electrode structural stability [14]. Thirdly and most important, “shuttle effect” caused by the LiPSs dissolution causes poor cycle stability, which obstructs the practical application of Li-S batteries [15].

Considerable research work has been made to tackle the aforementioned challenges. Based on the natural intrinsic conductivity and a stable skeleton structure, various porous carbon materials such as carbon nanospheres [16], graphene [17], carbon nanotubes (CNTs) [18], and carbide-derived carbons have been developed as cathodes for high performance Li-S batteries. Unfortunately, albeit the considerable progress, the physical interaction between the nonpolar carbon and LiPSs is too weak for sufficient sulfur fixation [16]. In view of this, polar materials such as metal oxides (MOs), metal sulfides (MSs), and metal-organic frameworks (MOFs) [19] have been extensively studied for their ability to form strong chemical bonds with LiPSs. For example, TiO_2_ [13], MnO_2_ [7], Al_2_O_3_ [18], ZnO [20], Co(OH)_2_ [21], and ZrO_2_ [22] showed excellent performance as sulfur host materials. As expected, both metal oxides and metal hydroxides as S host cathodes exhibit high discharge capacity and excellent cycling stability [23,24,25,26]. Compared with other metal (hydrogen) oxides, Ni(OH)_2_ has become a promising material due to its low cost and strong chemical bonding with LiPS. Zhang et al. use Ni(OH)_2_ nanoparticles as surface modifiers, a new type of Ni(OH)_2_ surface-modified C/S composites was prepared [27]. This modifier acts to strengthen the LiPS adsorption performance and inhibiting the migration of S, so that excellent electrochemical performance can be achieved. Xu et al. develop a three-dimensional porous hollow structure of Ni(OH)_2_ as the S host material [28], and the outer nanosheets endow it with large specific surface area and abundant active sites while acting as a conductive scaffold. Zhao et al. reported that rGO-coated Ni(OH)_2_ materials encapsulated sulfur nanoparticles could significantly improve the specific capacity and long-term cycling stability of lithium-sulfur batteries [29]. The introduction of porous CNT as a support material for LiPSs, not only improves the 3D conductive network structure, but also facilitates the electron transportation, resulting in high-rate capabilities [30]. Therefore, it is very promising to design a composite that decorates carbon nanotubes on nickel hydroxide nanosheets [31,32]. It can combine the advantages and alleviate the disadvantages of the two components [33,34].

Herein, we designed the CNTs reinforced hollow mesoporous Ni(OH)_2_ as a free-standing sulfur host matrix. In this structure, CNTs were grown on Ni(OH)_2_ nanosheets and wrapped active sulfur nanoparticles as an effective carrier to enable confinement shuttle effect whilst promoting their reaction kinetics. In addition, S/Ni(OH)_2_@CNT as the cathode for Li-S batteries, which have several apparent ad vantages. First, one-dimensional CNTs could provide fast electron transport paths [35], and hollow core acts as a nanoscale electrochemical reaction vessel that efficiently limits the large volume variation. Furthermore, the introduction of these abundant functional polar/hydrophilic groups of Ni(OH)_2_ nanosphere could restrict the “shuttle effect” of LiPSs through strong chemical adsorption [36,37,38,39,40]. Meanwhile, the intermediate thiosulfate generated by the reaction of nickel hydroxide and LiPSs can accelerate the redox kinetics and promote cycle stability [41,42]. As a result, the Li-S batteries assembled by Ni(OH)_2_@CNT cathode obtained excellent electrochemical performance, which opens up a new way for the research of cathode materials for Li-S batteries.

## 2. Experimental Section

### 2.1. Synthesis of Ni(OH)_2_@CNT and S/Ni(OH)_2_@CNT Composite

Materials: Ammonium hydroxide solution (28%, Aladdin, Shanghai, China), NiCl_2_·6H_2_O (99.9%, Aladdin, Shanghai, China), Urea (99.5%, Aladdin, Shanghai, China), Carbon nanotube dispersion (CNTs, 10 wt%, Aladdin, Shanghai, China).

Synthesis of Ni(OH)_2_@CNT composite: Using deionized water as the solution, configure 15 mL 0.2 M anhydrous nickel chloride and 15 mL 2 M urea solution. The two solutions were mixed to form a light green solution, which was stirred at a rotation speed of 300 r/min for 30 min. Under the strong stirring of 600 r/min, 6 mL of 13% aqueous ammonia solution was dropped into the above solution dropwise to form a blue solution. Take 400 mg of carbon nanotube dispersion (~10 wt%) and disperse it in 30 mL of absolute ethanol. The two solutions were uniformly mixed and then transferred to an autoclave for reaction at 120 °C for 12 h. Transfer the reacted solution into centrifuge tube, wash with deionized water, and centrifuge three times to remove soluble impurities [8]. The centrifuged product was collected to obtain Ni(OH)_2_@CNT.

Preparation of Sulfur-Based Composites: The S/Ni(OH)_2_@CNT composites were prepared by a melt impregnation method. First, sublimated sulfur was thoroughly mixed with the as-prepared Ni(OH)_2_@CNT at a mass ratio of 3:1, followed by heat treatment at 155 °C for 12 h under an inert atmosphere [43]. That is, S/Ni(OH)_2_@CNT is obtained. According to the SEM image shown in Figure S1, sublimation S is fully mixed with Ni(OH)_2_@CNT. Evenly distributed CNTs provide a guarantee for the electron transfer of the electrode. For comparison, S/Ni(OH)_2_ and S/CNT composites were prepared with Ni(OH)_2_ and CNT as sulfur host, respectively.

### 2.2. Materials Characterization

The surface morphology of the samples was examined using scanning electron microscopy (SEM, Sigma 500, Oberkochen, Germany) equipped with an EDS system. The hollow structure was also observed with high resolution transmission electron microscope (HRTEM, JEM-2100F, JEOL, Tokyo, Japan). X-ray diffraction (XRD) patterns of materials were acquired by Bruker D8 Discover (Karlsruhe, Germany) diffractometer with the 2θ of 10–60° using Cu-Kα radiation source. The content of sulfur was measured by Thermogravimetric Analysis (TGA, Perkin Elmer, Series7, Waltham, MA, USA) in N_2_ from 10 °C to 600 °C. The specific surface area and pore distribution calculation were measured by a V-Sorb 2800P analyzer instrument. The surface functional characteristics were analyzed by X-ray spectroscopy (XPS, ESCALAB 250Xi, Thermo Fisher Scientific, Waltham, MA, USA). The pore distribution of the samples was analyzed by Hg porosimetry by Mike Auto Pore IV 9500 (Norcross, GA, USA).

### 2.3. Electrochemical Measurements

The cathode was prepared by homogeneously mixing the S/Ni(OH)_2_@CNT composites, PVDF, and acetylene black with a mass ratio of 8:1:1. The discharge/charge capacity is provided by the active material (sulfur), and the test electrode contains about 0.8–1.0 mg cm^−2^ of the active material [44]. Lithium foils were used as the anode and porous polypropylene (Celgard 2300) was used as the separator. A UV-Vis spectrophotometer (Perkin Elmer) was adopted to analyze the adsorption capability of samples to Li_2_S_6_ [45]. At room temperature, a BTS4000 battery testing system (Neware, Shenzhen, China) was used to test the battery for galvanostatic discharge/charge performance. The electrochemical impedance spectroscopy (EIS) and cyclic voltammetry (CV) were both performed on a CHI-660E electrochemical workstation (Chenhua, Yangzhou, China).

### 2.4. Theoretical Calculations

To further verify the adsorption effect of Ni(OH)_2_ on LiPS, the binding energy (Ebind) between Ni(OH)_2_ and LiPS was calculated by density functional theory (DFT), calculated by the following equation [46]:

E_bind_ = E_total_ − E_LiPS_ − E_Ni(OH)_2__

where, E_total_, E_LiPS_ and E_Ni(OH)_2__ represent the energy of the corresponding substances. The magnitude of the binding capacity is positively correlated with the absolute value of E_bind_.

## 3. Results and Discussion

**Figure 1:**
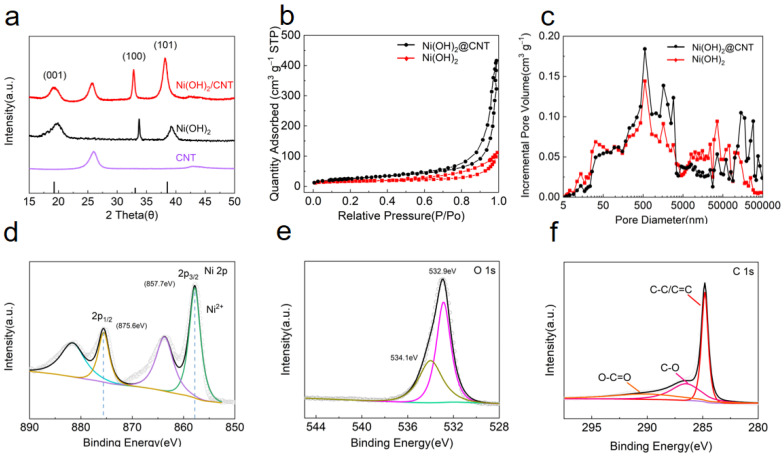
(**a**) XRD patterns of Ni(OH)_2_@CNT, Ni(OH)_2_ and CNT; (**b**) N_2_ adsorption-desorption isotherms of Ni(OH)_2_ and Ni(OH)_2_@CNT. (**c**) The pore size distribution of Ni(OH)_2_ and Ni(OH)_2_@CNT. The XPS spectra of (**d**) Ni 2p, (**e**) O 1s, and (**f**) C 1s of Ni(OH)_2_@CNT.

The XRD patterns of composites were displayed in Figure 1a. Both XRD patterns of the Ni(OH)_2_ and Ni(OH)_2_@CNT composite show three clearly characteristic peaks located at 19.8°, 34° and 39.1°, which correspond to (001), (100) and (012) lattice planes of Ni(OH)_2_ (JCPDS No.73-1520), respectively [47]. The sharp diffraction peaks demonstrated the good crystallinity of the as-synthesized Ni(OH)_2_ precursor. A typical peak at about 26° observed in CNT belongs to the (002) plane of carbon [48,49,50]. The crystal plane was basically in line with the standard card. As expected, it was suggesting that the carbon nanotube decorated Ni(OH)_2_ composites were prepared successfully.

Furthermore, the N_2_ adsorption-desorption isotherm was used to analyze pore characteristics (Figure 1b). Ni(OH)_2_@CNT exhibits a higher specific surface area than Ni(OH)_2_. In order to better understand the porosity of Ni(OH)_2_@CNT composite materials, the Hg porosity measurement method was used to analyze the pore size distribution of Ni(OH)_2_@CNT materials (Figure 1c). The pore volumes of Ni(OH)_2_ and Ni(OH)_2_@CNT composites are measured to be 3.37 cm^3^ g^−1^ and 3.71 cm^3^ g^−1^, respectively. Compared with Ni(OH)_2_, the Ni(OH)_2_@CNT composite has a higher macropore distribution. These results indicate that the large specific surface area and pore size distribution of Ni(OH)_2_@CNT composites satisfy the premise of high sulfur loading. In addition, the hollow structure and outer nanosheets of Ni(OH)_2_@CNTs can provide abundant pores and larger active interfaces, enabling faster ion/electron transportation and facilitating redox reactions.

To further validate the chemical states of Ni(OH)_2_@CNT composite, XPS measurements were performed. The positions of all peaks are calibrated with the binding energy of 284.8 eV reported in the literature to standardize the energy of the instrument [51,52]. The high-resolution spectrum of Ni 2p (Figure 1d), showing two spin-orbit peaks and their satellite peaks. The presence of Ni(OH)_2_ could be proved by the two spin-orbit peaks locating at 875.6 eV (Ni 2p_1/2_) and 857.7 eV (Ni 2p_3/2_). The Ni 2p spectrum was mainly composed of Ni^2+^, which could inhibit the dissolution of LiPSs through chemical interaction with LiPSs. In the O 1s spectrum (Figure 1e), the peaks at 532.9 eV and 534.1 eV correspond to metal-OH bonds and C=O groups, respectively [53]. The three sub-peaks of the C 1s XPS spectrum (Figure 1f) are located at 284.8 eV, 286.51 eV and 290.21 eV, corresponding to C-C/C=C, C-O and O-C=O bonds, respectively. As shown in Figure S2a, all the elements of S, Ni, and O were detected by XPS on S/Ni(OH)_2_@CNT. The Ni 2p spectrum (Figure S2b) displays four main peaks, which are in accord with the Ni 2p in Ni(OH)_2_@CNT. The deconvolution of the S 2p_3/2_ spectrum (Figure S2c) revealed peaks located at 162.9 eV, 164.2 eV and 169.5 eV, which could be assigned to S^2−^, S^0^ and SO_x_^2−^ species, respectively. For the O 1s (Figure S2d), an intense band at 531.7 eV is obviously observed, which is considered to be the O from the OH^−^. A low peak at 533.2 eV can be observed in the O 1s spectrum due to the influence of carbonate ions and hydroxyl groups.

**Figure 2:**
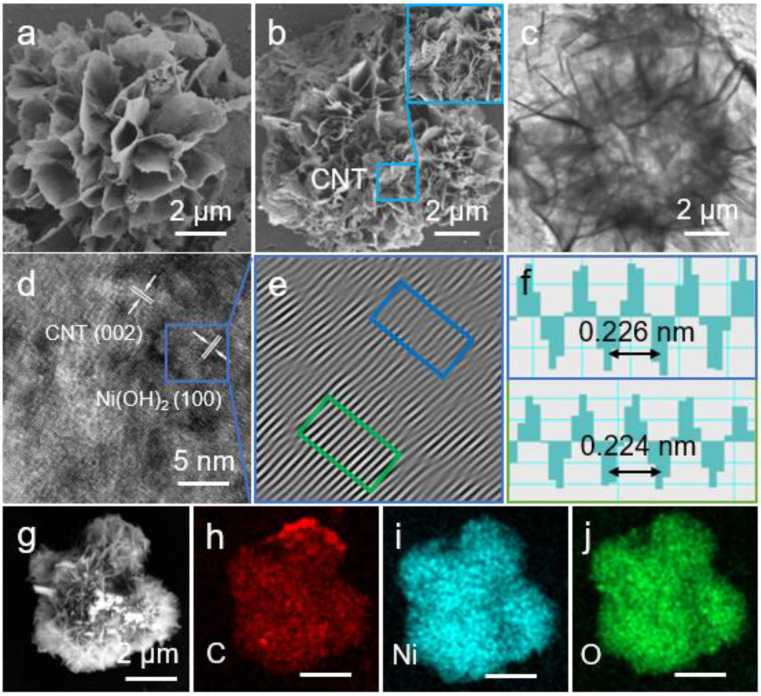
SEM image of Ni(OH)_2_ (**a**); SEM and TEM images of Ni(OH)_2_@CNT (**b**,**c**); (**d**) HRTEM image of Ni(OH)_2_@CNT; (**e**,**f**) inverse FFT pattern and the corresponding lattice spacing profiles; (**g–j**) elemental mapping of Ni(OH)_2_@CNT.

According to the SEM image shown in Figure 2a, the synthesized Ni(OH)_2_ exhibited a spherical morphology. SEM image demonstrated that the nanosheets are uniformly tightly anchored onto the surface of the nanoscale hollow spheres. This structure effectively avoided the loss of LiPSs. At the same time, the close contact of the nanosheet shell provided a guarantee for electron transfer of the electrode. The SEM image of the Ni(OH)_2_@CNT obtained was shown in Figure 2b. It can be clearly seen that the spherical structure of composite was not impaired and the smooth surfaces became rough, which may be caused by the CNTs. As shown in the TEM image (Figure 2c), the Ni(OH)_2_@CNT nanosheets possessed abundant void spaces, which can provide rich chemical reaction active sites. The crystalline feature of the Ni(OH)_2_@CNT was further proved by HRTEM. The (100) plane of Ni(OH)_2_ shown in Figure 2d was established by inverse fast Fourier transform (IFFT) patterns. Figure 2e,f shows the IFFT lattice image, where the fringe spaces of selected areas respectively are 0.226 nm (blue frame) and 0.224 nm (green frame), corresponding to the (100) planar spacing of Ni(OH)_2_. Furthermore, elemental mapping images manifested the elemental homogeneous distribution of Ni, O and C in Ni(OH)_2_@CNT (Figure 2g–j).

**Figure 3:**
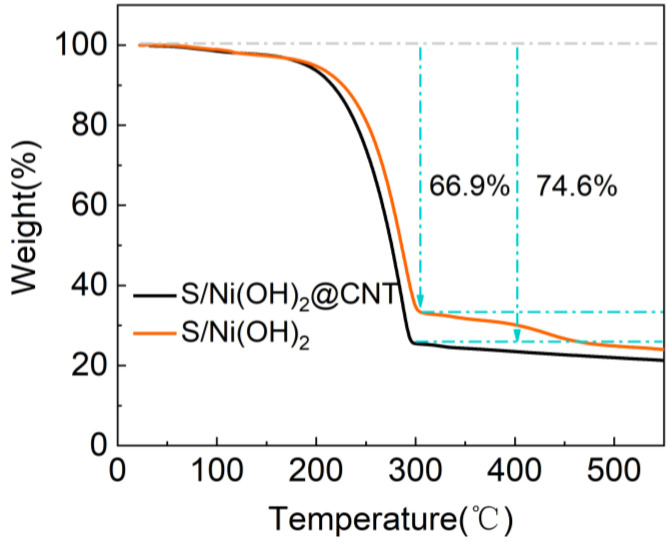
TGA curves of S/Ni(OH)_2_ and S/Ni(OH)_2_@CNT.

TGA was employed to analyze the sulfur content in composites (Figure 3). Three distinct phases of weightlessness are observed on the TGA plots. The first major weightlessness cause of the evaporation of embedded water molecules at around 50 °C. The following loss occurs for the transformation from Ni(OH)_2_ to NiO at ~220 °C. The third weightlessness occurred when NiO_2_ is reductive decomposed at high temperatures above 400 °C. Due to the low content of Ni(OH)_2_ in S/Ni(OH)_2_@CNT, the loss was relatively small, which can be neglected. TGA plots shows an extremely fast weight loss between 160 and 300 °C, corresponding to the rapid sublimation of sulfur in the composite. The sulfur content in the S/Ni(OH)_2_@CNT and S/Ni(OH)_2_ composites is determined to be about 74.6 wt% and 66.9 wt% through the TGA, respectively. Under the same S: Samples ratio, the S content obtained in the experiment of this paper has a higher loading (Table S1, Supporting Information).

**Figure 4:**
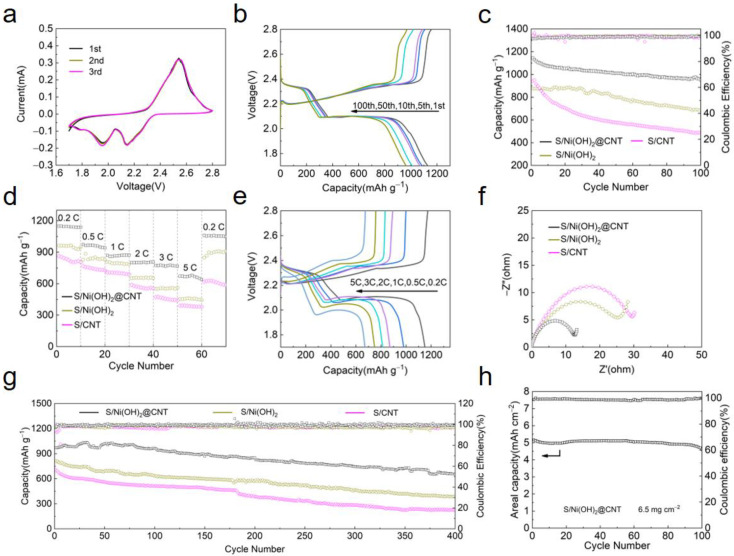
(**a**) Cycle voltammograms of Li-S batteries with S/Ni(OH)_2_@CNT cathodes; (**b**) discharge/charge curves at 0.2 C; (**c**) Cycling performance with different electrodes at 0.2 C; rate capability (**d**,**e**) profiles of S/Ni(OH)_2_@CNT cathode at various current rates between 0.2 and 5 C; (**f**) EIS spectra of batteries with the different electrodes; (**g**) long term cycling performance of Li-S batteries with different electrodes at 1 C for 400 cycles; (**h**) cyclic stability of S/Ni(OH)_2_@CNT cathode with high sulfur loading of 6.5 mg cm^−2^ at 0.2 C.

All electrochemical performance tests use CR2032-type coin cells. As shown in Figure 4a, one oxidation peak and two reduction peaks appear in the CV curves. In the cathodic reaction, the peak located at 2.14 V represented the formation of soluble polysulfides (Li_2_S_n_, 4 ≤ n ≤ 8), the reduction of LiPSs to low-order LiPSs were associated with the cathodic peak at around 1.96 V. During the anodic cycle, only a sharp peak was shown at ~2.56 V, which is attributed to the chemical transformation from Li_2_S to sulfur [54,55]. The nearly overlapped CV plots in the three cycles for the S/Ni(OH)_2_@CNT indicated its highly electrochemical stability. The sharp and overlapped peaks indicated the high conductivity, high reaction stability and fast kinetics granted by S/Ni(OH)_2_@CNT electrodes. Figure 4b displayed the discharge/charge curves for the S/Ni(OH)_2_@CNT electrodes. Typical reaction plateaus were shown and were identical with the CV curves. The hollow structure and high conductivity of the S/Ni(OH)_2_@CNT electrode rendered small polarization and high sulfur utilization. Moreover, the S/Ni(OH)_2_@CNT electrode exhibits an initial specific capacity of 1146 mAh g^−1^ at 0.2 C with an initial coulombic efficiency of 98.1%. The cycling performance of different electrodes at 0.2 C is shown in Figure 4c. S/Ni(OH)_2_@CNT exhibited an initial discharge capacity of 1146 mAh g^−1^, indicating a good sulfur utilization. After 100 cycles, the capacity retention of S/Ni(OH)_2_@CNT cathode is 83.4%, which is larger than S/Ni(OH)_2_ (689 mAh g^−1^) and S/CNT (483 mAh g^−1^) electrodes.

The S/Ni(OH)_2_@CNT also delivered excellent rate performance (Figure 4d). After initial activation, the S/Ni(OH)_2_@CNT electrode delivers 1148 mAh g^−1^ at 0.2 C. At 0.5, 1, 2, 3, and 5 C, the reversible capacities of Li-S batteries with the S/Ni(OH)_2_@CNT electrode were around 974, 869, 801, 773, and 672 mAh g^−1^, respectively. Moreover, when the discharge/charge rate was abruptly returned to 0.2 C, the capacity of S/Ni(OH)_2_@CNT cathode recovered to 1054 mAh g^−1^, confirming the outstanding cycling stability and structure robustness of the electrode. It was noteworthy that S/Ni(OH)_2_@CNT cathode displayed a small plateau potential difference (∆E) at all current densities (Figure 4e).

Electrochemical impedance spectra of the cells with S/CNT, S/Ni(OH)_2_ and S/Ni(OH)_2_@CNT electrodes were conducted to investigate the difference of internal resistance, as shown in Figure 4f. Three Nyquist plots of the S/Ni(OH)_2_@CNT, S/Ni(OH)_2_, and S/CNT batteries were in the same shape consisting of one semicircle referring to the charge-transfer resistance (R_ct_) and one sloped line associated with Li^+^ diffusion process. The S/Ni(OH)_2_@CNT cathode shows the lowest charge transfer resistance value (R_ct_ = 13 Ω) when compared with S/Ni(OH)_2_ (R_ct_ = 25 Ω) and S/CNT (R_ct_ = 30 Ω), indicating that the battery with S/Ni(OH)_2_@CNT cathode had the fastest charge transfer kinetics, which can be ascribe to the introduction of the S/Ni(OH)_2_@CNT electrodes into Li-S batteries greatly enhanced the electronic conductivity and reaction kinetics.

Long-term cycling test at 1 C was further performed on the S/Ni(OH)_2_@CNT, S/Ni(OH)_2_ and S/CNT cathode (Figure 4g). The S/Ni(OH)_2_@CNT provided a high capacity of 972 mAh g^−1^ at 1 C. The discharge capacity exhibited an evident increase in the first 50 cycles. The S/Ni(OH)_2_@CNT cathode was gradually infiltrated by the electrolyte, so the active material can be effectively utilized. After 400 cycles, the discharge capacity was 652 mAh g^−1^, which meant the capacity loss per cycle was only 0.081%.

To demonstrate the potential of the S/Ni(OH)_2_@CNT in the high energy density Li-S battery, the cycling property of Li-S Batteries with high sulfur loadings was tested. As shown in Figure 4h, the S/Ni(OH)_2_@CNT electrode exhibits a stable high areal capacity of 4.6 mAh cm^−2^ and an energy density of 1679.4 Wh Kg^−1^ (Figure S3) even at a high sulfur loading of 6.5 mg cm^−2^. Besides, the performance advantages of as-developed S/Ni(OH)_2_@CNT electrode can also be confirmed from the comparison with recently reported carbon-based electrodes (Table S1, Supporting Information).

**Figure 5:**
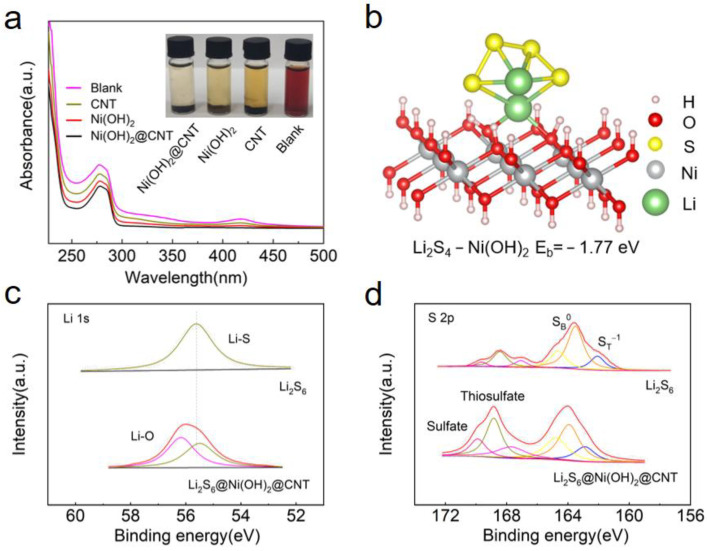
(**a**) Optical observation of LiPS adsorption by CNT, Ni(OH)_2_, Ni(OH)_2_@CNT and the corresponding UV–vis spectra. (**b**) optimized configuration and the corresponding binding energy of Li_2_S_4_ on Ni(OH)_2_@CNT surfaces. (**c**) Li 1s and (**d**) S 2p spectra of Li_2_S_6_ before and after adsorbed on Ni(OH)_2_@CNT.

Generally speaking, if the Li_2_S_2_ and Li_2_S cannot be converted completely and quickly at a high rate, they would gradually deposit on the surface of the electrode and block the reaction channel between electrons and lithium ions. The Ni(OH)_2_ with a nano-sheet structure can accelerate the diffusion of electrons/lithium ions, while preventing the polysulfides from dissolving during the cycling process. Among the various LiPSs, trapping the Li_2_S_6_ and Li_2_S_4_ species within the cathode matrix was crucial. To intuitively verify the adsorption ability of Ni(OH)_2_ to LiPSs, the polysulfide (Li_2_S_6_) adsorption tests have been carried out on the Ni(OH)_2_@CNT. As the inset in Figure 5a, 50 mg of Ni(OH)_2_@CNT, Ni(OH)_2_ and CNT were placed in a THF solution containing 0.05 M Li_2_S_6_, respectively. After standing for 12 h, the color of the Li_2_S_6_ solution with Ni(OH)_2_@CNT became colorless to further illustrate the affinity of Ni(OH)_2_@CNT for LiPSs. According to the UV-vis spectra, in the polysulfide solution, S_6_^2−^ had a characteristic peak, located at 278 nm. Obviously, after adding Ni(OH)_2_@CNT to the simulated solution, the intensity of the S_6_^2−^ peak dropped sharply. Ni(OH)_2_@CNT demonstrated a remarkable ability to absorb Li_2_S_6_, which is consistent with the above observation.

To further confirm the strong chemisorption of Ni(OH)_2_ on LiPS, the binding energy of Li_2_S_4_ on Ni(OH)_2_ was studied using density functional theory (DFT) calculations. Figure 5b shows the geometrically stable configuration of Li_2_S_4_ adsorbed on Ni(OH)_2_, in which there is a “lithium bond”-like bridge. Adsorption configurations of Li_2_S_4_ on Ni(OH)_2_ were showed with a binding energy of −1.77 eV, which indicates that Ni(OH)_2_ presents significantly adsorption capacity for LiPS. Moreover, the interaction between Ni(OH)_2_@CNT and LiPS Ni(OH)_2_@CNT was also studied by XPS analysis. The peak at 56.2 eV of the Li_2_S_6_@Ni(OH)_2_@CNT indicates the formation of Li-O bonds (Figure 5c). Figure 5d shows the S 2p spectrum of bare Li_2_S_6_, where the two pairs of peaks at 162.1 and 163.5 eV correspond to the terminal S and bridging S, respectively. After interaction with Ni(OH)_2_@CNT, these peaks showed a considerable shift to the higher binding energy (BE) range, indicating that the electron cloud density of sulfur atoms decreases after adsorption of Ni(OH)_2_@CNT. The result demonstrated that Ni(OH)_2_@CNT had strong chemical and physical absorbing ability, which inhibit the effects of LiPSs, and were significant to the confinement of the shuttle effect.

## 4. Conclusions

In summary, we report a Li-S battery using Ni(OH)_2_@CNT with hierarchical intersecting hollow structures as sulfur carriers. First, it accelerates electron/lithium–ion diffusion and prevents LiPSs from dissolving during the cycle. A cooperative interface is composed of “physical confinement” and “chemical adsorption” to effectively capture LiPSs and facilitate the reaction kinetics. Furthermore, the introduction of these abundant functional polar/hydrophilic groups of Ni(OH)_2_@CNT could restrict the shuttle effect of LiPSs through strong chemical adsorption. Finally, the compact structure of the S/Ni(OH)_2_@CNT cathode keeps the sulfur safely confined to micro-pores. This work sheds light on the promising practical applications of transition metal hydroxide in Li-S batteries.

## Data Availability

Data are contained within the article.

## Supplementary Materials

The following are available online at https://www.mdpi.com/article/10.3390/nano12050886/s1, Figure S1. SEM images of S/Ni(OH)_2_@CNT; Figure S2. (a) XPS survey and high-resolution (b) Ni 2p, (c) S 2p, and (d) O 1s spectra of S/Ni(OH)_2_@CNT; Figure S3. Energy density of S/Ni(OH)_2_@CNT cathode with high sulfur loading of 6.5 mg cm^−2^ at 0.2 C; Table S1. Sulfur loading comparison of S/Ni(OH)_2_@CNT with previously reported S/C cathodes; Table S2. Performance comparison among different C-based sulfur electrodes. References [56,57,58,59,60,61,62,63,64,65,66] were cited in the supplementary materials.
