# Supplementary material for: Carbon Nanotube-Modified Nickel Hydroxide as Cathode Materials for High-Performance Li-S Batteries
Source: Nanomaterials (Basel). 2022 Mar 7;12(5):886. doi: 10.3390/nano12050886 (PMC8912414; doi:10.3390/nano12050886)
Supplement: Supplementary file 1 [file nanomaterials-12-00886-s001.zip › nanomaterials-1518002-supplementary.pdf]

nanomaterials

Supporting Information

Carbon Nanotube-Modified Nickel Hydroxide as Cathode Materials for High-Performance Li-S Batteries

Qianwen Jin [1], Yajing Yan [1], Chenchen Hu [1], Yongguang Zhang [1,*], Xi Wang [2,*] and Chunyong Liang [1,*]

[1] State Key Laboratory of Reliability and Intelligence of Electrical Equipment, Hebei University of Technology, Tianjin 300130, China; jinqianwen111@163.com (Q.J.); yajingy@126.com (Y.Y.); h15364966537@163.com (C.H.)

[2] China Center for Information Industry Development, Beijing 100048, China

* Correspondence: yongguangzhang@hebut.edu.cn (Y.Z.); liangchunyong@hebut.edu.cn (C.L.); wangxi@ccid-thinktank.com (X.W.)

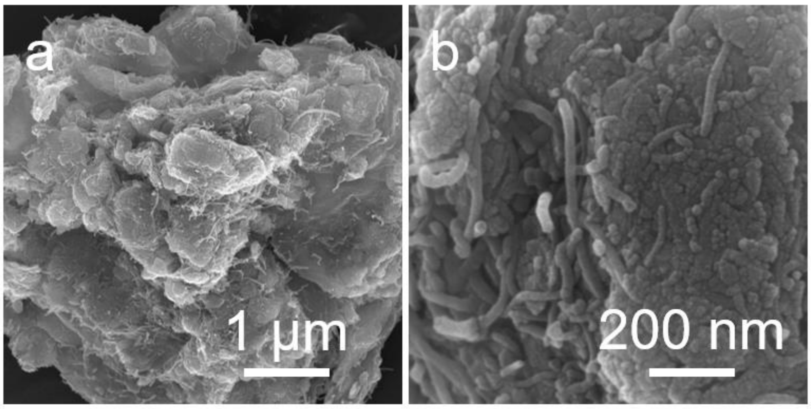

Figure S1. SEM images of S/Ni(OH)2@CNT.

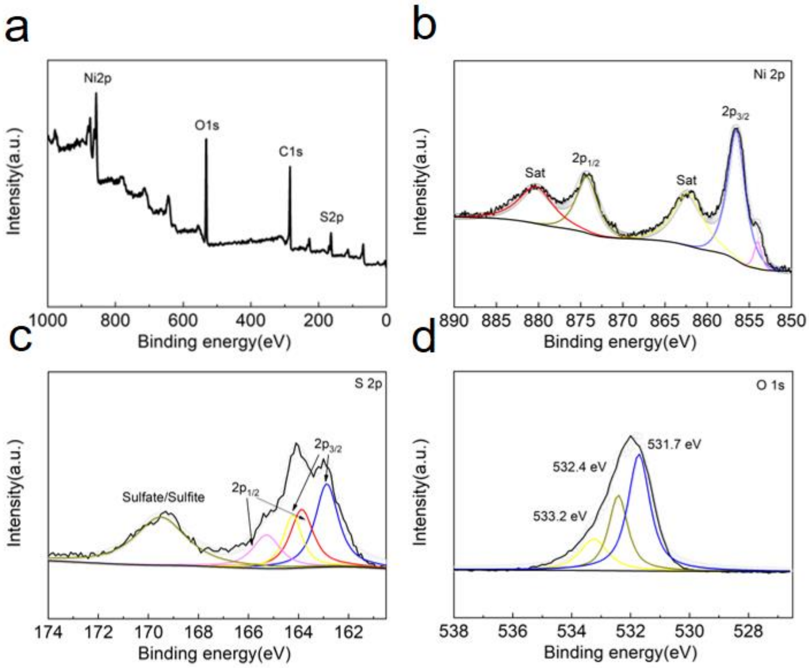

Figure S2. (**a**) XPS survey and high-resolution (**b**) Ni 2p, (**c**) S 2p, and (**d**) O 1s spectra of S/Ni(OH)2@CNT.

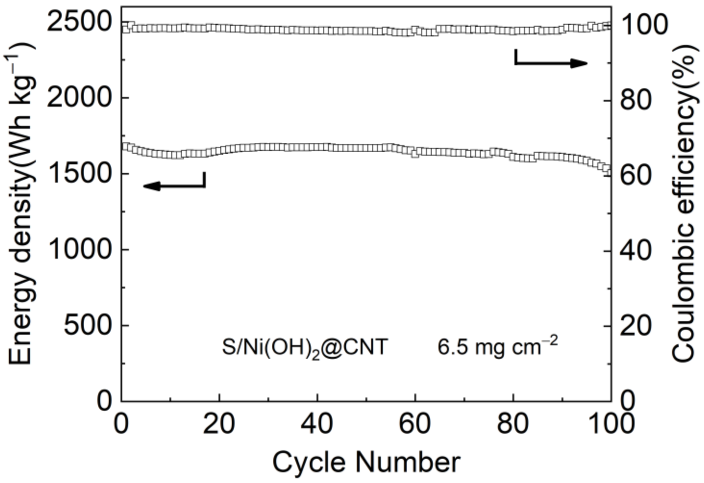

Figure S3. Energy density of S/Ni(OH)$_2$@CNT cathode with high sulfur loading of 6.5 mg cm^{-2} at 0.2 C.

Table S1. Sulfur loading comparison of S/Ni(OH)$_2$@CNT with previously reported S/C cathodes.

Samples	Weight Ratio (S:Samples)	Sulfur Content (%)	Ref.
DIB@CNT	3:1	67.0	[1]
CNT/ CPO/CPNC-1	7:3	70.0	[2]
CNTs	5:1	78.0	[3]
CNT@UIO66-SH	3:1	60.0	[4]
CCB	3:2	59.3	[5]
MgAl-LDH@CNT	9:11	55.0	[6]
CNTs/(Ni-P)	7:3	70.8	[7]
**Ni(OH)$_2$@CNT**	**3:1**	**74.6**	**This work**

Table S2. Performance comparison among different C-based sulfur electrodes.

Samples	Initial Capacity (mAh g^{-1})/rate	Areal Capacity (mAh cm^{-2})	Cycle	S Loading (mg cm^{-2})	Ref.
NSHC/S	1586/0.1C	4.07	100	6.72	[8]
S-C@S	958/0.1C	1.72	200	2.9	[9]
CP/Fe–N-GMOC/S	1473/0.2C	5	120	6	[10]
S@SPPC	1386/0.1C	4.4	150	4.8	[11]
S/3DOM ZnO	1110/0.2C	4.5	100	5	[12]
**S/Ni(OH)$_2$@CNT**	**1146/0.2C**	**4.6**	**100**	**6.5**	**This work**

References

1. Hu, G.J.; Sun, Z.H.; Shi, C.; Fang, R.P.; Chen, J.; Hou, P.X.; Liu, C.; Cheng, H.M.; Li, F. A Sulfur-Rich Copolymer@CNT Hybrid Cathode with Dual-Confinement of Polysulfides for High-Performance Lithium-Sulfur Batteries. *Adv. Mater.* **2017**, *29*, 1603835.
2. Song, Z.C.; Lu, X.L.; Li, X.Y.; Jiang, N.; Huo, Y.; Zheng, Q.J.; Lin, D.M. Tailored multifunctional hybrid cathode substrate configured with carbon nanotube-modified polar $Co(PO_3)_2$/CoP nanoparticles embedded nitrogen-doped porous-shell carbon polyhedron for high-performance lithium-sulfur batteries. *J. Colloid Interface Sci.* **2020**, *575*, 220–230.
3. He, J.R.; Bhargav, A.; Yaghoobnejad Asl, H.; Chen, Y.F.; Manthiram, A. 1T′-ReS_2 Nanosheets In Situ Grown on Carbon Nanotubes as a Highly Efficient Polysulfide Electrocatalyst for Stable Li-S Batteries. *Adv. Energy Mater.* **2020**, *10*, 2001017.
4. Liu, X.; Wang, S.; Wang, A.L.; Wang, Z.N.; Chen, J.; Zeng, Q.H.; Chen, P.P.; Liu, W.; Li, Z.X.; Zhang, L.Y., A new cathode material synthesized by a thiol-modified metal-organic framework (MOF) covalently connecting sulfur for superior long-cycling stability in lithium-sulfur batteries. *J. Mater. Chem. A.* **2019**, *7*, 24515–24523.
5. Niu, X.Q.; Wang, X.L.; Wang, D.H.; Li, Y.; Zhang, Y.J.; Zhang, Y.D.; Yang, T.; Yu, T.; Tu, J.P. Metal hydroxide - a new stabilizer for the construction of sulfur/carbon composites as high-performance cathode materials for lithium-sulfur batteries. *J. Mater. Chem. A.* **2015**, *3*, 17106–17112.
6. Hwang, J.-Y.; Kim, H.M.; Shin, S.; Sun, Y.-K. Designing a High-Performance Lithium-Sulfur Batteries Based on Layered Double Hydroxides-Carbon Nanotubes Composite Cathode and a Dual-Functional Graphene-Polypropylene-Al_2O_3 Separator. *Adv. Funct. Mater.* **2018**, *28*, 1704294.
7. Wu, Y.Q.; Luo, Z.X.; Wang, X.R.; Fu, G.L.; Lei, W.X.; Zou, Y.L.; Yin, B.B.; Ma, Z.S.; Pan, Y.; Jiang, W.J. Cotton-like CNTs/(Ni-P)/S composites with enhanced electrochemical performance of lithium-sulfur battery. *Mater. Res. Bull.* **2022**, *145*, 111529.
8. Tong, Z.M.; Huang, L.; Guo, J.Y.; Gao, Y.; Zhang, H.J.; Jia, Q.L.; Luo, D.; Lei, W.; Zhang, S.W. Simultaneously achieving fast sulfur redox kinetics and high-loading in lithium–sulfur batteries. *Carbon* **2022**, *187*, 451–461.
9. Chen, J.S.; Liu, Y.; Liu, Z.K.; Chen, Y.; Zhang, C.G.; Yin, Y.N.; Yang, Q.L.; Shi, Z.Q.; Xiong, C.X. Carbon nanofibril composites with high sulfur loading fabricated from nanocellulose for high-performance lithium-sulfur batteries. *Colloid. Surface. A* **2020**, *603*, 125249.
10. Li, H.; Liu, D.; Zhu, X.X.; Qu, D.Y.; Xie, Z.Z.; Li, J.S.; Tang, H.L.; Zheng, D.; Qu, D.Y. Integrated 3D electrodes based on metal-nitrogen-doped graphitic ordered mesoporous carbon and carbon paper for high-loading lithium-sulfur batteries. *Nano Energy* **2020**, *73*, 104763.
11. Zhao, T.Q.; Tan, X.H.; Song, L.T.; Guo, L.M.; Liu, Y.L.; Kang, X.H.; Meng, X.M.; Wang, H.F.; Chu, W.G. Up-Scalable Conversion of White-Waste Polystyrene Foams to Sulfur, Phosphorus-Codoped Porous Carbon for High-Performance Lithium–Sulfur Batteries. *ACS Appl. Energy Mater.* **2020**, *3*, 9369–9378.
12. Han, H.S.; Wang, T.; Zhang, Y.G.; Nurpeissova, A.; Bakenov, Z. Three-Dimensionally Ordered Macroporous ZnO Framework as Dual-Functional Sulfur Host for High-Efficiency Lithium-Sulfur Batteries. *Nanomaterials* **2020**, *10*, 2267.
